# Explanation of the influence of geomorphometric variables on the landform classification based on selected areas in Poland

**DOI:** 10.1038/s41598-024-56066-6

**Published:** 2024-03-05

**Authors:** Krzysztof Dyba

**Affiliations:** Applied Geoinformatics Research Unit, Adam Mickiewicz University, Bogumiła Krygowskiego 10, 61-680 Poznań, Poland

**Keywords:** Environmental sciences, Geomorphology

## Abstract

In recent years, automatic image classification methods have significantly progressed, notably black box algorithms such as machine learning and deep learning. Unfortunately, such efforts only focused on improving performance, rather than attempting to explain and interpret how classification models actually operate. This article compares three state-of-the-art algorithms incorporating random forests, gradient boosting and convolutional neural networks for geomorphological mapping. It also attempts to explain how the most effective classifier makes decisions by evaluating which of the geomorphometric variables are most important for automatic mapping and how they affect the classification results using one of the explainable artificial intelligence techniques, namely accumulated local effects (ALE). This method allows us to understand the relationship between predictors and the model’s outcome. For these purposes, eight sheets of the digital geomorphological map of Poland on the scale of 1:100,000 were used as the reference material. The classification results were validated using the holdout method and cross-validation for individual sheets representing different morphogenetic zones. The terrain elevation entropy, absolute elevation, aggregated median elevation and standard deviation of elevation had the greatest impact on the classification results among the 15 geomorphometric variables considered. The ALE analysis was conducted for the XGBoost classifier, which achieved the highest accuracy of 92.8%, ahead of Random Forests at 84% and LightGBM at 73.7% and U-Net at 59.8%. We conclude that automatic classification can support geomorphological mapping only if the geomorphological characteristics in the predicted area are similar to those in the training dataset. The ALE plots allow us to analyze the relationship between geomorphometric variables and landform membership, which helps clarify their role in the classification process.

## Introduction

Geomorphology is a scientific discipline that studies landforms, their features and the processes that shape them^1^. One of the key aspects of geomorphology is the mapping process, which involves identifying landforms and determining their spatial distribution in the context of processes occurring on the Earth's surface^2^. Traditional and automatic mapping are two different approaches to mapping landforms based on their features, shape, and spatial distribution.

Traditional geomorphological mapping is based on fieldwork and manual interpretation of various data sources (for example, digital elevation models, topographic maps, aerial or satellite imagery), which requires a high level of expertise and experience. Therefore, this approach is time consuming and expensive. Another debatable issue is the repeatability of mapping results related to the subjective nature of interpretation, which can consequently lead to different divisions and ranges of landforms or soil units^3^.

On the other hand, automatic geomorphological mapping can be more efficient and cheaper, and most importantly, can provide reproducible results by removing the aspect of subjectivity. Basically, three different approaches to automatic classification can be distinguished, i.e., the pixel-based^4–8^, object-based^9,10^ and pattern-based. The first two are currently used as state-of-the-art, but the last one is new and requires further research.

The pattern approach mainly relies on convolutional neural networks (CNNs), which involve a multi-step learning process using convolutional layers to create a feature map that extracts certain image patterns. CNNs have become very popular in computer vision due to their high efficiency in identifying low-level features and patterns, making them very effective for data classification^11,12^.

Recent research on the application of convolutional neural networks in geomorphology includes the use of a multi-channel deep neural network architecture to classify landforms^13^, a comparison of Random Forests and U-Net models to classify loess formations^14^, a comparison between traditional and automated U-Net-based approaches^15^, and classification using textural properties of the terrain^16^.

So far, several initiatives have been undertaken to develop high-resolution digital geomorphological maps of selected areas in Poland based on traditional mapping, including Roztocze Upland^17^, Pomeranian and Warmian–Masurian voivodeships^18^, Mazovia^19^, Carpathians^20^, Narew National Park^21^, Wielkopolska–Kujawy Lowland, Mysliborsk Lakeland and Szczecin Lowland^22^, Podlasie^23^, and Tykocin^24^. Nevertheless, the mentioned studies were conducted by independent research teams and are not unified, thus they have different catalogs of landforms, mapping principles and spatial scales.

However, research on automatic classification of the geomorphological landforms in Poland remains at an early stage. The first study compares unsupervised automatic classification with the traditional mapping for the Sudetes^25^. The second study also concerns unsupervised classification for the area of the Silesian Upland^26^. Another study on supervised classification was conducted by Janowski et al.^27^, in which the authors compared machine learning algorithms for classifying glacial landforms in the Lubawa Upland and Gardno–Leba Plain areas using ground truth dataset. In a previous article co-written by the present author, we clustered the landforms of the entire country using an unsupervised method^28^. This means that we made no prior assumptions about geomorphological units. Finally, we separated 20 land surface types in the process of interpreting and labeling clusters.

The first objective of this article is to perform a supervised classification using machine learning based on the available sheets of the digital geomorphological map of Poland. Unlike the unsupervised approach, the catalog of geomorphological units is known in advance, but the problem is to map it as best as possible using an automatic classification method. The second objective is to interpret the classification decisions made by the model, in particular to explain which geomorphometric variables are most relevant and how they affect the classification results.

## Materials and methods

We divided this section into several subsections to clearly present the extensively used materials and methods. Section “Digital geomorphological map” describes the digital geomorphological map of Poland. Section “Morphometric variables” provides information on geomorphometric explanatory variables and how they are processed. Section “Selection of a classification model” presents the machine learning and neural network models employed, while Section “Validation” presents the methods and metrics for their validation. Section “Model explanation” describes the method to explain the classifier's decision. Finally, Section “Software” contains technical information about the software used.

### Digital geomorphological map

**Figure 1:**
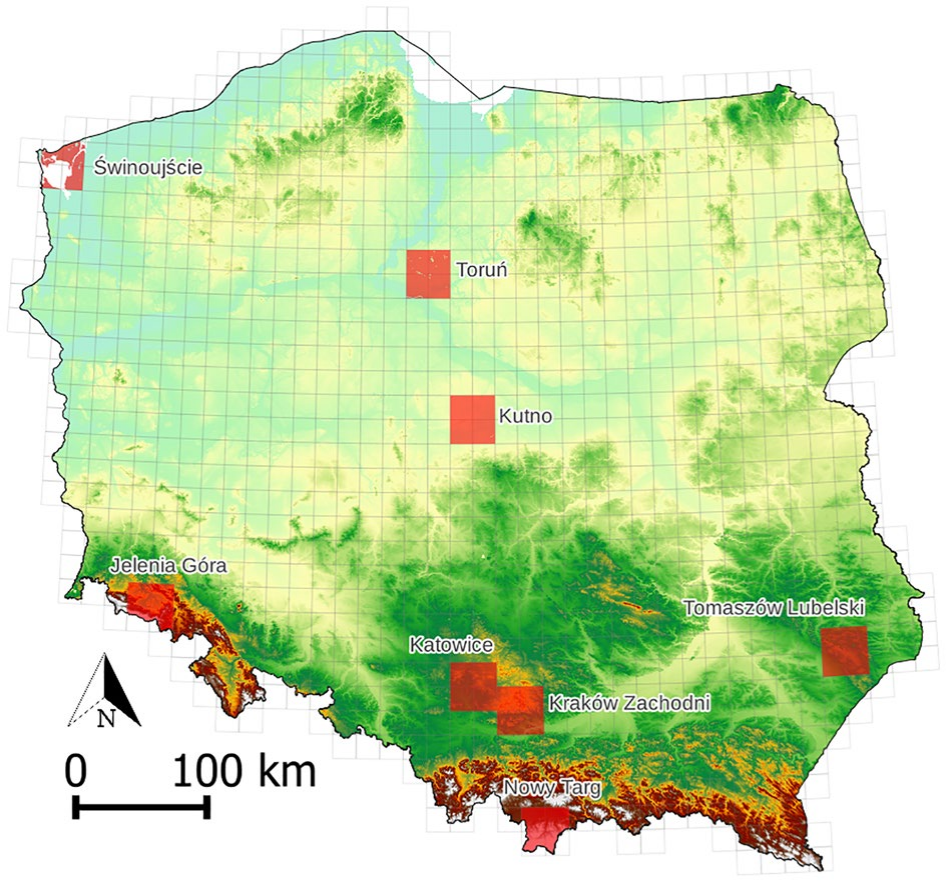
Sheet coverage of the digital geomorphological map of Poland.

The digital geomorphological map of Poland on the scale of 1:100,000 is a vector map showing the forms of relief and the genesis of the Earth's surface alongside information about its formation^29^. The color scheme is based on the Gustavsson et al.^30^ concept with modifications. Eight available sheets with a total area of 9072 km^2^ were used as a reference dataset (Fig. 1). Currently, it is the only such detailed and up-to-date source on a national scale with uniform principles of development. The landforms presented are from all morphogenetic zones, including the coastal area (Świnoujście), the young and old glacial areas (Toruń and Kutno), the upland areas (Katowice, Kraków Zachodni and Tomaszów Lubelski), and the areas of young and old mountains (Jelenia Góra and Nowy Targ). In the technical manual there are 77 surface divisions in 10 morphogenetic groups; however, only 54 divisions can be found on the available sheets. The landforms are listed in Supplementary Fig. S1.

**Figure 2:**
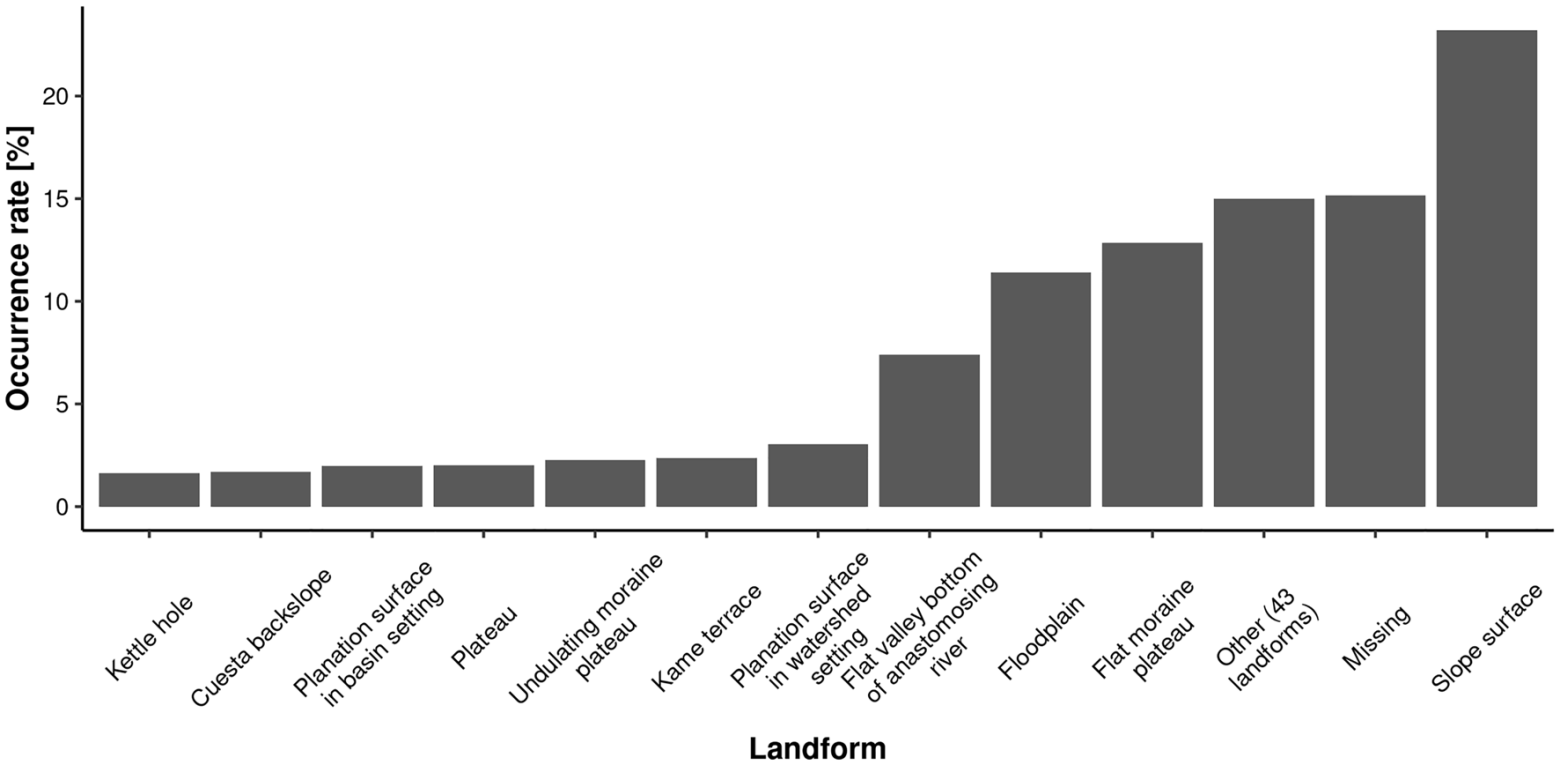
Distribution of the geomorphological forms from the used sheets. All forms with a total area of less than 166 km^2^ (i.e., the 80th percentile) are combined into one category in the figure: Other.

The representativeness of the morphological forms is strongly unbalanced; for instance, the slope surface landform accounts for more than 23% of the total dataset, while the other 43 landforms represent less than 15% (Fig. 2). This issue is a major problem in automatic classification methods. This means that the algorithm is unable to learn how to correctly classify forms that are a significant minority (permille) in the dataset. To address this problem, we reduced the size of the 14 largest classes to 150,000 observations using the data under-sampling procedure^31^ and removed the two least numerous classes (beach and dune plain). The second issue relates to missing values (*NA*) that result from areas not covered by mapping or water surfaces (Fig. 2). In the case of machine learning algorithms, typically missing values can be omitted (they will not be included in the training set), while neural networks use them in the learning process, and then they are masked (excluded). The final dataset consisted of over 3.3 million observations (pixels).

Machine learning algorithms require a discrete representation of data, for this reason we rasterized vector maps to a resolution of 30 m in the Polish geodetic coordinate system 1992 (EPSG: 2180). For this purpose, we created a classification table that contained the original category names encoded as text and their corresponding IDs in numerical form. We coded missing data (*NA*), water reservoirs, and areas not surveyed with a value of 0.

### Morphometric variables

**Figure 3:**
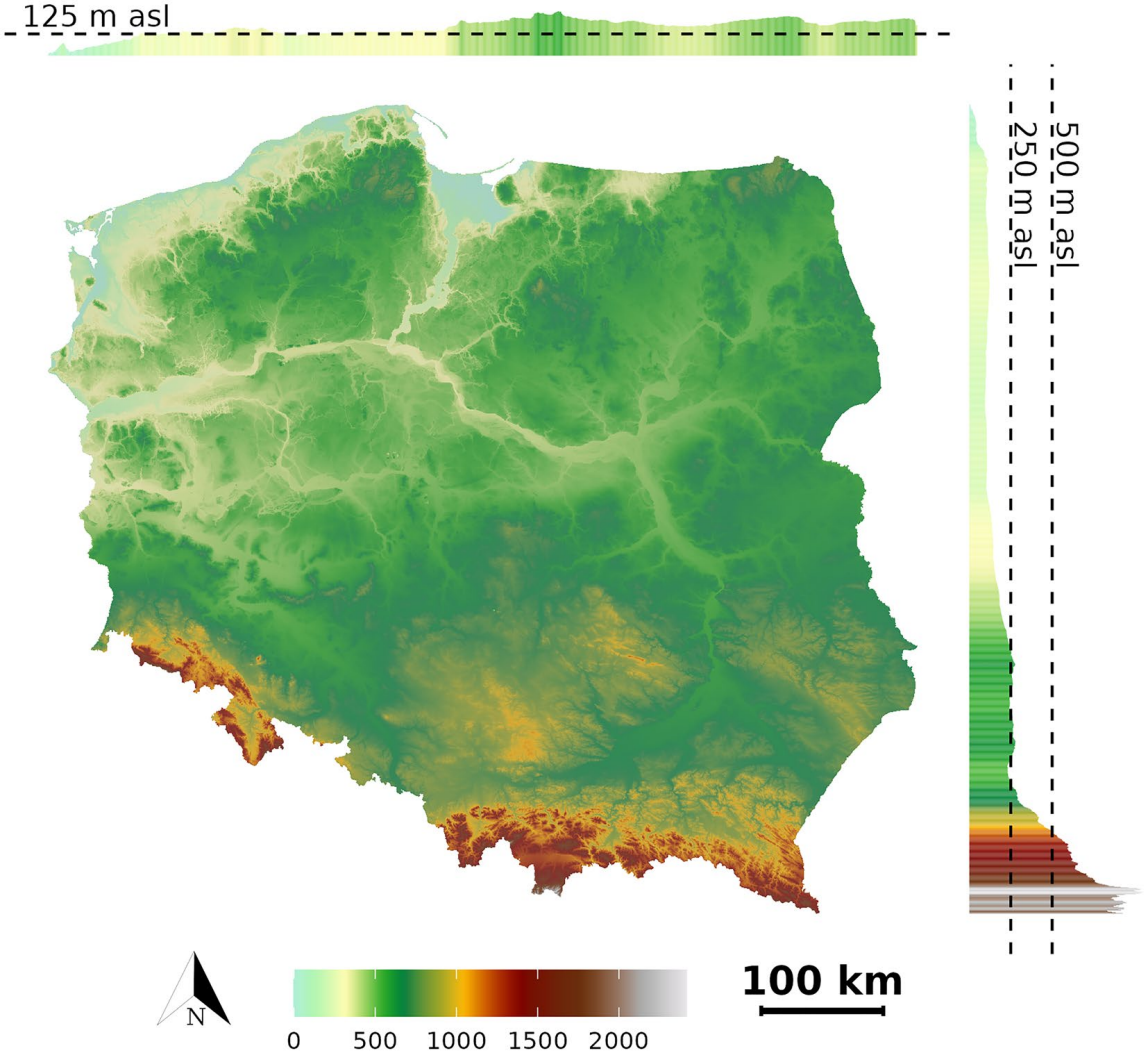
Elevation map of Poland with hillshading. Histograms with average elevation values calculated for latitudes and longitudes are seen on the sides.

As the main data source, we used a digital terrain model with a resolution of 30 m adapted from Digital Terrain Elevation Data Level 2 (Fig. 3). The data has been smoothed and resampled, so the artifacts (noise strips) seen in the original do not appear^32^. Then we generated a number of derivative products based on it.

**Figure 4:**
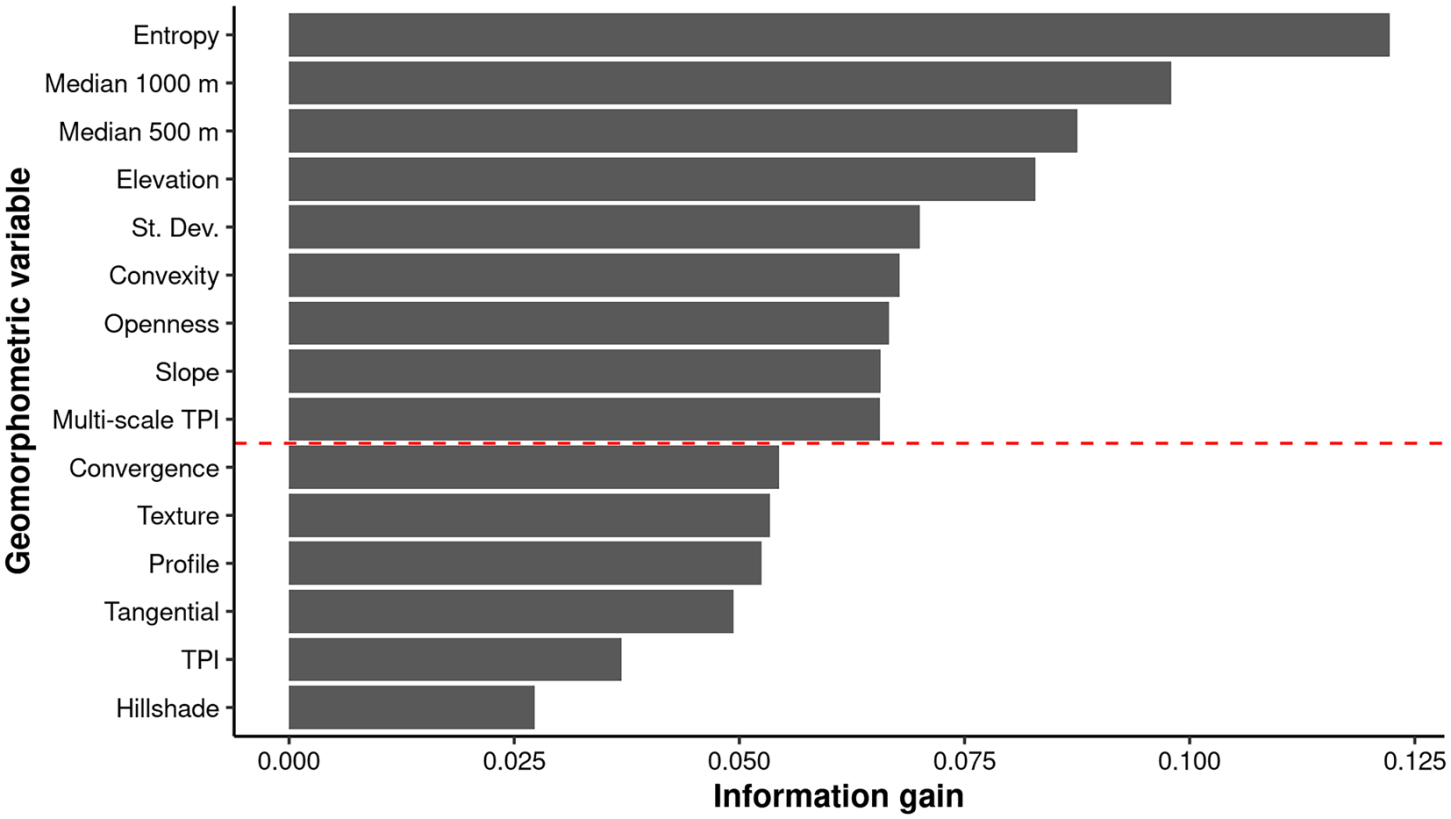
Importance of the geomorphometric variables for the geomorphological classification using the XGBoost model. The higher the value, the greater the suitability. The red dashed line indicates discarded low-significance variables.

More than 100 different geomorphometric variables can be found in popular applications for geomorphometric analysis. It is impossible to include all of them for technical reasons (hardware limitations, processing time) and analytical reasons (some are strongly correlated). Therefore, we considered the 15 most commonly used and made a final selection of the most important features for classification using model performance metrics, visual inspection and model information gain (Fig. 4). Eventually, we reduced their number to 9 (Table 1), which increased the performance of the classifier (i.e., faster training and prediction, and reduced memory consumption) and, most importantly, simplified the structure of the model, thus making its decisions easier to interpret. We initially tested absolute elevation, multidirectional hillshade^33^, slope^34^, topographic position index^35^, multi-scale topographic position index, tangential and profile curvatures^34^, convergence index with radius^36^, terrain surface texture^6^, terrain surface convexity^6^, topographic openness^37^, aggregated elevation, local standard deviation, and textural features including contrast, energy and entropy^38^.

**Table 1:** Morphometric variables used in this study.

#	Variable	Range	Mean value	Unit
1	Absolute elevation	− 0.03, 2483	171 ± 129.2	m
2	Slope	0, 76.1	1.75 ± 3.08	deg
3	Local standard deviation 1000 m	0, 262.7	5 ± 8.3	m
4	Multi-scale topographic position index	− 53.1, 44.6	0 ± 0.7	m
5	Terrain surface convexity	0, 88.6	48.7 ± 7.5	–
6	Entropy	7, 940318	52,441 ± 44,508	–
7	Topographic openness	0.61, 1.7	1.55 ± 0.03	–
8	Median elevation 500 m	0, 2335	171 ± 128.9	m
9	Median elevation 1000 m	0, 2238	170 ± 128.5	m

**Figure 5:**
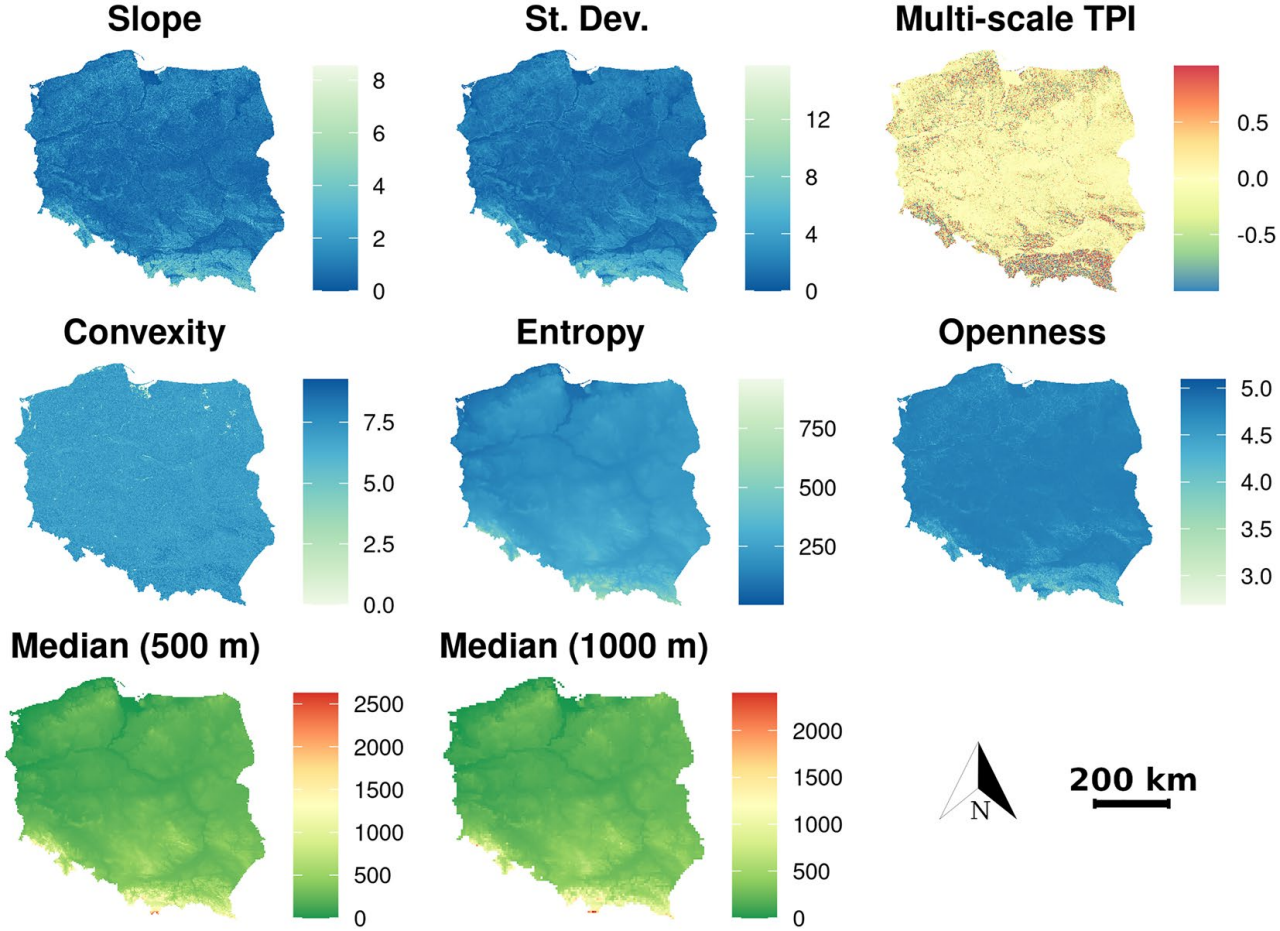
Geomorphometric variables used in this study. The absolute elevation is shown in Fig. 3 with the topographic color scale. The aggregated by median elevations of 500 and 1000 m look almost identical, but actually represent different spatial scales. The slope, standard deviation, convexity, entropy, openness variables are scaled by the square root, and the multi-scale TPI by the sine to better represent spatial variability.

The aggregated elevation was calculated using a statistic (in this case, the median) from neighboring pixels at a lower spatial resolution (500 and 1000 m respectively), and then the aggregated cell was divided into smaller blocks corresponding to a resolution of 30 m. If it was possible to set the analysis radius, we set it to 16 pixels (representing an area of about 0.7 km^2^). Additionally, we removed the variables above a linear correlation of 0.9 because they essentially convey the same information except for aggregated elevation and entropy (they are perfectly correlated with absolute elevation but contain information on a larger spatial scale), which allows for mapping morphological objects with continuity. The geomorphometric variables used in this study are presented in Fig. 5.

### Selection of a classification model

We compared the three most popular models based on machine learning—Random Forests^39^ and gradient boosting including XGBoost^40^ and LightGBM^41^, and the convolutional neural network (CNN) model—U-Net^42^. The main difference between random forests and gradient boosting is that the former reduces the variance of a large number of complex models with low bias (the models are built independently and parallelly), while the gradient boosting reduces the bias of a large number of simple models with low variance (the models depend on each other because each is based on all previous small models with the appropriate weight, hence the name “boosting”). Both XGBoost and LightGBM models are based on gradient boosting, but the former uses an “exact” algorithm, while the latter uses an “approximate” algorithm (observations with similar values are aggregated into bins). This acts as a compromise between performance and accuracy of trained models.

In contrast, convolutional neural networks are primarily dedicated to computer vision, whereas machine learning models focus on modeling tabular data. They consist of multiple layers that can extract vital image features (such as edges) and reduce the spatial resolution while retaining the important information. Thus, it can be expected that the recognition of spatial patterns will be independent of the shift (i.e., the model will be able to recognize the same pattern in a different place) and the spatial hierarchy of objects will be considered (for example, the first layer of the network will learn to recognize small local patterns, and the next layer will aggregate them into larger structures). In the case of machine learning, this is not directly possible, and the data must be prepared in an appropriate way (feature engineering).

#### Model hyperparameters tuning

Machine learning models require a predeterminating of the hyperparameters such as maximum tree depth, number of leaves (nodes), learning rate, etc. to be effective. This procedure is called tuning. In order to find the most effective hyperparameters, we used a random search procedure, which involves defining a search space (combination) of hyperparameters and random sampling. Then, using the drawn combination, the model is trained, and its performance is evaluated on an independent dataset.

We considered the following hyperparameters in this procedure: *eta* (step size shrinkage used to prevent overfitting), *max_depth* (maximal tree depth), *nrounds* (number of iterations), *subsample* (subsample ratio of the training data) in XGBoost; *num.trees* (number of trees to grow), *mtry* (number of variables randomly sampled as candidates at each node split), *min.node.size* (minimal node size), *max.depth* in Random Forests; *learning_rate* (performs the same function as *eta* in XGBoost), *max_bin* (maximum number of bins that feature values will be bucketed in), *num_leaves* (maximum number of leaves in one tree), *nrounds*, *max_depth, bagging_fraction (*performs the same function as *subsample* in XGBoost*), feature_fraction* (ratio of variables randomly sampled for each tree) in LightGBM. Appropriate selection of these hyperparameters prevents the model from overfitting the training data.

It should be emphasized that the applied machine learning algorithms do not use the pixel neighborhood, so information about the shape and continuity of geomorphological forms is not included. In fact, information about the values of the geomorphometric variables is only used for individual pixels. We used feature engineering, to address this problem, which is based on three elements: 1) calculation of the geomorphometric variables in the local window (if it was possible); 2) use of selected geomorphometric variables at lower spatial resolution to detect larger landforms; 3) use image textural features. See Section “Morphometric variables” for more details.

#### Convolutional neural network

We evaluated the convolutional neural networks using the U-Net model in Tensorflow^43^. This architecture consists of two main components, i.e., a contracting path (encoder) and an expansive path (decoder). The former progressively reduces the spatial resolution of the input image while increasing the number of features. The expansive path is the inverse of the contracting path and involves upsampling operations to restore the spatial resolution and reduce the number of features. The final layer consists of a convolutional layer with a softmax activation function, producing pixel-wise class predictions. The detailed architecture is shown in the original article by Ronneberger et al. in Fig. 1^42^.

Several tile sizes were tested as input, i.e., 32 × 32, 64 × 64, 128 × 128 and 256 × 256 pixels. Finally, the most satisfactory results were obtained for blocks of 128 × 128 pixels due to the compromise between capturing spatial patterns by the model and the number of missing values in the tiles. To solve the problem of a large percentage of missing data, we removed those blocks for which the number of missing values was more than 70%, ultimately resulting in a total of 685 raster blocks. In order to increase the amount of input data, data augmentation was applied by flipping images in the vertical and horizontal planes. Adam's algorithm was used as the optimization function^44^. It should be noted that deep learning models have hundreds of thousands of parameters for tuning and, therefore, require much more input data compared to machine learning models. In this study, U-Net was used only as a reference method and its lower performance is expected compared to the other models tested.

### Validation

We used holdout validation to validate the results—30% of the randomly selected input dataset was used as a test set to calculate the models' performance metrics, i.e., accuracy, Cohen's kappa coefficient (κ) and Matthews correlation coefficient (φ). The former provides overly optimistic results for unbalanced datasets, but the second and third are corrected for this effect and offer more reliable results. However, because we under-sampled classes to balance our dataset, the difference between these metrics is insignificant. Moreover, we used fivefold cross-validation to test the accuracy of the most efficient classifier (i.e., XGBoost) in this study for individual areas. Note that non-spatial validation can produce somewhat biased results^45,46^, and in order to evaluate the performance completely independently, new geomorphological sheets (i.e., those that have not been used to train the models) should be used.

### Model explanation

The models used in this study are black box models. This means that the predictions and decisions they generate are not interpretable in a simple way. In other words, the high complexity of the algorithms causes difficulties in explaining how it actually works^47,48^. In order to understand which geomorphometric features the model uses to make decisions, we used the XGBoost gain metric (Fig. 4), which determines the improvement in model performance by adding a specific feature to the decision tree. Moreover, in addition to examining which variables are most useful for mapping, we also evaluated the interactions between the classification results and each geomorphometric variable using accumulated local effects plots^49^.

The accumulated local effect (ALE) is a machine learning interpretability method that allows gain insights into the model's behavior, identifying how features affect predictions. The ALE method is similar to the partial dependence plot^50^, but is faster and more robust (i.e., it enables an analysis of the correlated variables). The former focuses on local effects that are calculated in small windows, while the PDP calculates average values. The resulting ALE plot shows how the model prediction changes as the particular feature value increases (assuming that the other features are fixed), enabling an examination of the relationship between a feature and the model's prediction. In practice, this helps identify interactions that are not evident by simply assessing the significance of the features like using XGBoost gain metric. To the best of the author's knowledge, this method has not been previously used to explain the decision making of the classification models in geomorphological mapping.

### Software

The geomorphometric variables were generated in GRASS GIS 7.8.0^51^ with default function parameters. The data analysis and machine learning parts were completed in R^52^, while the neural networks were used in Python. In particular, the *stars* package was utilized for processing the raster data^53^, and *sf* for the vector data^53^. Statistical metrics were implemented in the *yardstick*^54^ package. The *ranger* package was used to train Random Forests^55^. Accumulated local effects plots were generated by the *ALEPlot* package^49^.

The development of the models was very time-consuming. It took nearly two weeks of continuous computations to train all 1180 models on an AMD Ryzen 9 5900X with 128 GB RAM. The models were trained in parallel on 12 physical CPU cores. The largest part of the trained models were models based on LightGBM algorithm, because of the largest number of hyperparameters to be tuned compared to other machine learning models.

## Results

### Classification

As a result of the evaluation on an independent test dataset, the XGBoost model proved to be the best with an accuracy of 92.8%. It was followed by Random Forests with an accuracy of 80.4% and LightGBM with an accuracy of 73.7%. The worst performance was achieved by the U-Net model due to insufficient training data (Table 2). During model tuning, the highest performance of the classifiers was obtained for the following hyperparameters:

1. XGBoost: *eta* = 0.2; *max_depth* = 20; *nrounds* = 150; *subsample* = 0.6
2. Random Forests: *num.trees* = 1000; *mtry* = 5; *min.node.size* = 1; *max.depth* = 20
3. LightGBM: *learning_rate* = 0.05; *max_bin* = 2048; *num_leaves* = 70; *nrounds* = 150; *max_depth* = 15; *bagging_fraction* = 1; *feature_fraction* = 1

**Table 2:** Evaluation of the model classification performance.

Model	Accuracy	Kappa coefficient	Matthews correlation coefficient
Random Forests	0.840	0.830	0.830
XGBoost	0.928	0.917	0.917
LightGBM	0.737	0.724	0.725
U-Net	0.598	0.576	0.576

The higher the metric values, the better the model.

Further performance of the models can be improved by using larger values for the *max_depth* and *max_bin* hyperparameters, but this actually results in overfitting on the test dataset.

The potential application of model fusion may be intriguing. This technique typically results in an overall improvement in classification performance using aggregated results from several different models. However, this is provided that all models offer similar and high prediction efficiency, which is not case in this study. Ultimately, this would reduce the quality of the prediction and, moreover, it would become impossible to explain the performance of the combined models.

**Figure 6:**
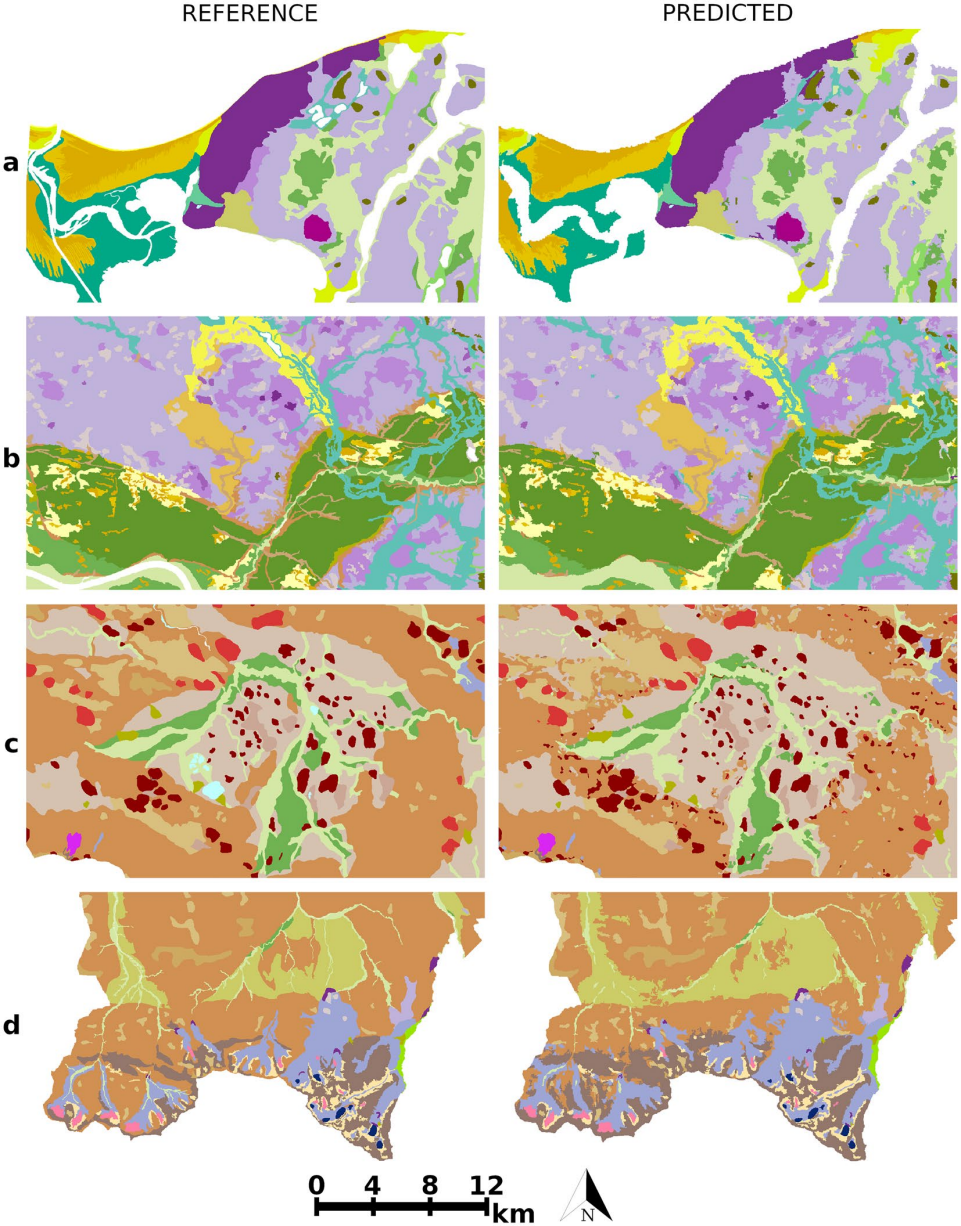
Comparison of the reference data (left) with predicted landforms (right) in the area of: (**a**) Wolin Island; (**b**) Chełmno-Dobrzyń Lakeland; (**c**) Jelenia Góra Basin; (**d**) Tatra Mountains. The predicted raster was smoothed with a modal filter of 5 pixels and landforms smaller than 21 pixels (~ 18,000 m^2^) were removed using a sieve filter. The legend is available in Supplementary Fig. S1.

From this point on, only the XGBoost classifier is subjected to further analysis because it achieved the best result compared to the other models. The analysis of models with lower performance is unjustified, especially in the context of explaining how geomorphometric variables influence landform classifications (i.e., misclassifications mean misinterpretations). In the evaluation of the predicted landforms on individual sheets, XGBoost recorded the best accuracy for Jelenia Góra at 96.2%, and the lowest for Tomaszów Lubelski at 88% (Table 3). The average accuracy value using cross-validation was over 93%, while the Kappa coefficient and Matthews correlation coefficient values were slightly lower. This demonstrates the high potential application, provided that the predicted landforms and distributions of geomorphometric variables are similar in both the test and training datasets. However, it is not possible to conclude that there is a strong correlation between the number of landforms and model accuracy—classification performance is rather related to the representativeness of the forms and the complexity of the spatial patterns associated with the geomorphological characteristics of the areas. Examples of the classifier's application are shown in Fig. 6. It is noteworthy that small landforms appear on the predicted rasters that are not visible on the geomorphological map. This may be due to the higher spatial resolution of the geomorphometric variables compared to the reference map (not necessarily the prediction errors).

**Table 3:** Performance of the XGBoost models for individual sheets based on fivefold cross-validation.

Sheet	Mesoregion	Number of landforms	Accuracy	Kappa coefficient
Świnoujście	Szczecin Coastland	19	0.961	0.957
Toruń	Chełmno–Dobrzyń Lakeland, Toruń–Eberswalde Ice Marginal Valley	25	0.913	0.908
Kutno	Central Masovia Lowland, Southern Wielkopolska Lowland	15	0.958	0.952
Jelenia Góra	Western Sudety, Mountains	19	0.962	0.958
Tomaszów Lubelski	Roztocze Upland, Sandomierz Basin	15	0.880	0.866
Katowice	Silesia Upland, Woźniki–Wieluń Upland	13	0.943	0.936
Kraków	Krakow–Częstochowa Upland, Kraków Gate	17	0.949	0.945
Nowy Targ	Orawa–Podhale Basin, Tatra Range	16	0.940	0.934

The physico-geographical mesoregions are defined based on Solon et al.^56^ classification.

### Model explanation

We first assessed the overall relevance of each geomorphometric variable for mapping. Among the tested variables, entropy, median elevation and absolute elevation turned out to be the most significant for classification. Next ranked were standard deviation, convexity and openness, slope and multi-scale TPI. The least useful for mapping were texture, profile and tangential curvatures, TPI, and hillshade (Fig. 4). The last group of variables with the lowest importance was excluded from the final classification since they do not actually improve mapping results, but significantly increase processing time, require additional memory and cause greater model complexity.

**Figure 7:**
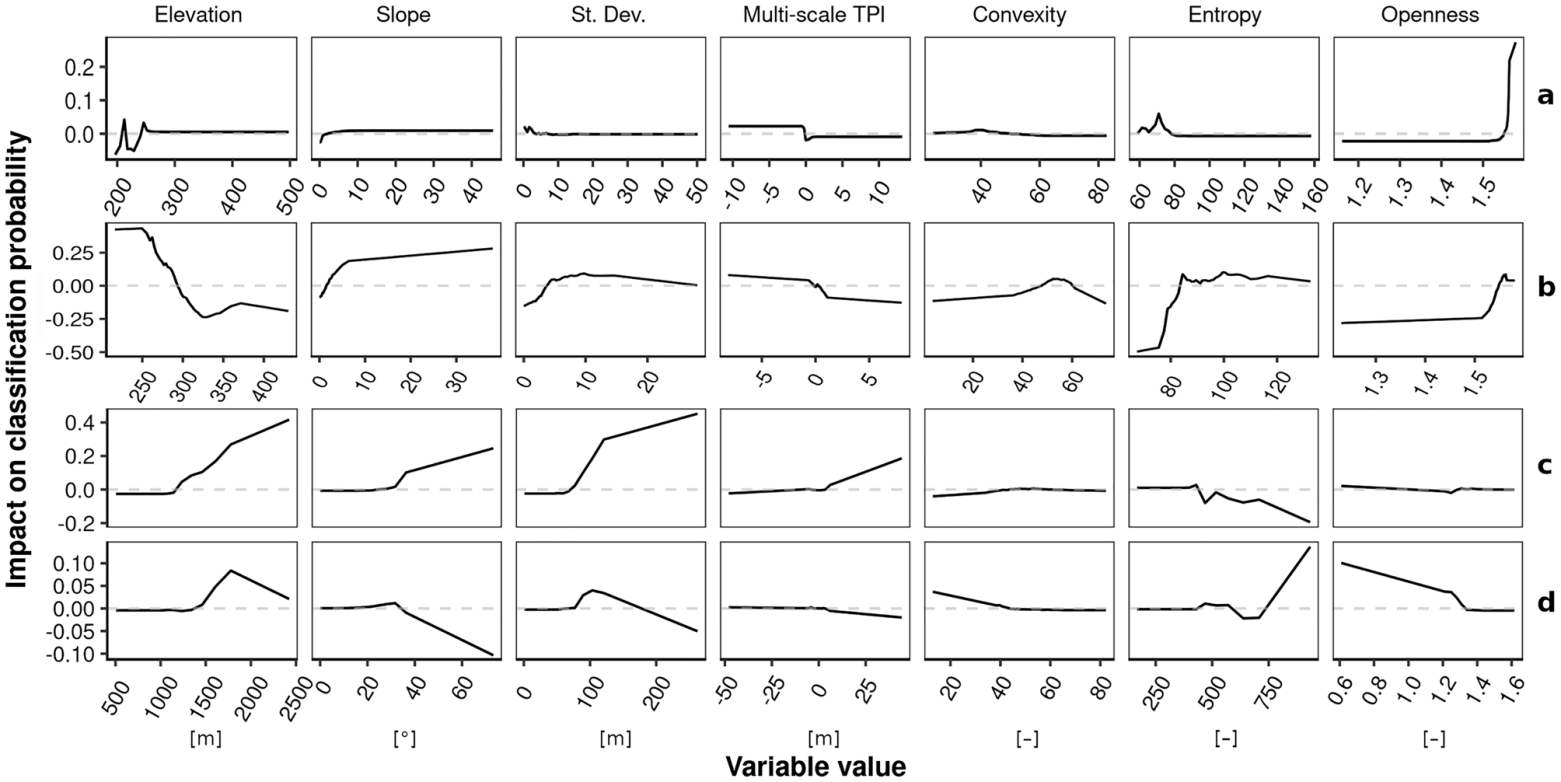
Accumulated local effects plot showing how geomorphometric variables affect the probability of classifying: (**a**) proluvial plain; (**b**) plateau; (**c**) rock wall/rock slope; (**d**) depositional scree slope. Entropy is expressed in thousands.

In the next step, we deepened the analysis of the relationship between the used geomorphometric variables, and the probability of the landform specified with accumulated local effects. As an example, we chose the four well-representative landforms, for instance: (a) proluvial plain; (b) plateau; (c) rock wall/rock slope; (d) depositional scree slope (Fig. 7). All other geomorphological landforms are presented in Supplementary Fig. S2.

Figure 7 shows how the probability of landform affiliation changes depending on geomorphometric variables. The first landform proluvial plain (Fig. 7a) is an extensive sandy flat surface created as a result of the periglacial-fluvial accumulation process. It is noticeable, in this case, the greatest impact on the detection of this form is the openness feature, whose high values (above 1.55) indicate an open and flat surface. The other geomorphometric variables are not very significant. The second example is the plateau (Fig. 7b), which is usually characterized by an irregular surface and explicit hillsides. In this case, low values of two features, i.e., entropy (below 85,000) and openness (below 1.58), reduce the probability of classifying this form, while an increase in the value of the slope increases this probability (in particular, a slope above 10°). The last example is rock wall/rock slope (Fig. 7c) and depositional scree slope (Fig. 7d). The former is a very steep or rugged fragment of the surface with a high slope, in which the process of weathering and falling rock materials occurs, creating an accumulated rubble slope at the foot of the slope. The latter usually takes the form of a mound or heap that is composed of rock rubble from a rock wall/rock slope. To detect the rock wall/rock slope, the slope, standard deviation and multi-scale TPI variables are important, high values of which increase the probability of classifying this landform. However, in the case of the depositional scree slope, high values of slope (above 30°) and standard deviation (above 150 m) reduce the probability of classifying this landform. The probability of classifying this landform by the model increases with high entropy (above 750,000) and low values of openness (below 1.3), which is probably related to the size of the rock material that creates irregular (undulated) surfaces. These examples demonstrate the convergence of classification decisions made by the model and geomorphological knowledge.

**Figure 8:**
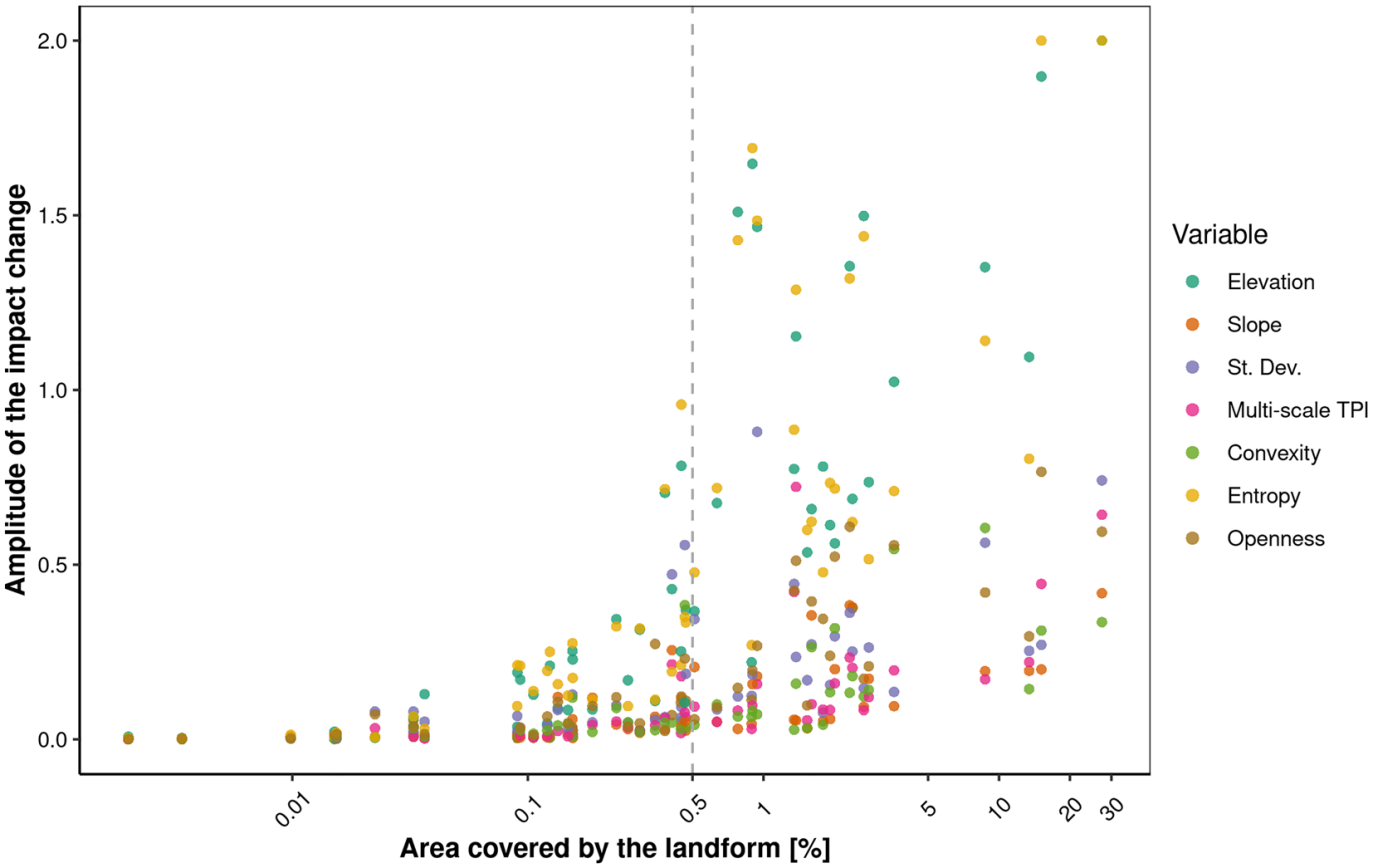
Amplitude of the impact change on the landform classification depending on its area and geomorphometric variables. Each set of points represents a different landform. 0.5% is the threshold value (marked with a dashed gray line) at which there is a significant increase in amplitude. The values on the X-axis are presented on a logarithmic scale. The total area is 9072 km^2^.

We also considered how the landform area represented in the dataset relates to the variability of the impact on the classification probability calculated from accumulated local effects. For this purpose, we defined the amplitude as the difference between the influence that increases the probability of being classified in a given class (the maximum is 1) and the influence that reduces this probability (the minimum is − 1), so the maximum amplitude can be 2. We noticed a positive relationship between this amplitude and the area of the landforms (Fig. 8). This means that it is easier for the model to provide a classification decision when the sample is larger. The largest amplitude occurs for the elevation, entropy and standard deviation, and this is consistent with the variable importance results from the XGBoost model. Moreover, it should be emphasized that the range of impact between sheets is different. This is because the sheets present areas of varying morphogenesis with different levels of geomorphological features (marking); therefore, some geomorphometric variables are more (or less) useful for characterizing the forms occurring there. In practice, this means that if the slope surface class can be easily classified on the Toruń sheet (i.e., a young glacial area) using the slope variable, it may be impossible on the Nowy Targ sheet (i.e., a young mountains area) due to the completely different structure and characteristics of the Tatra Mountains range.

## Discussion

As demonstrated, the accumulated local effect plot is a valuable tool for interpreting the classification decisions made by the machine learning model. Surprisingly, to the best of the author's knowledge, this technique has not yet been used in geomorphological mapping. Fundamentally, it allows us to check why the classifier has distinguished a given landform, which is especially important in the case of incorrect classifications (we can interpret on the basis of explanatory variables what causes the error). Moreover, this method can be used even in traditional mapping; if a geomorphologist is not sure about recognizing a certain landform in the field, he can assist with ALE plots. Its main advantage is the relative ease of interpretation since it provides a clear visualization of how each geomorphometric feature influences the model's predictions. In this study, it was used to interpret the classification decisions based on the gradient boosting model (XGBoost), but actually it can be applied to a wide range of different models (for example, linear models, tree-based models, neural networks). While ALE is certainly a useful tool, it also has some drawbacks. The main limitations are related to small datasets, low feature variability and the sensitivity of the model itself. The quality of the input dataset and the accuracy of the model should be carefully considered before conclusions are drawn.

Comparing the obtained results for convolutional neural networks, we can see differences in the accuracy of the classifiers between those provided by Du et al.^13^: 83–98%, Li et al.^14^: 78–87%, Meij et al.^15^: 44–94%, Xu et al.^16^: 84% and 70%, and in this study (59.8%). These differences are due to two reasons. The first is that in the mentioned studies, classifications were carried out only for a few geomorphological units, while in this study, 54 different landforms were classified. Naturally, this means obtaining such high-performance values is more complicated. The second reason is the selection of the research area. The areas chosen by the cited authors are very diverse and relatively easy to distinguish, which does not entirely illustrate the scale of the problem. The largest challenge is the analysis of the areas of complex genesis with poorly marked geomorphological features. In this case, the lower efficiency of the classifier is expected for the Polish sheets used, which consist mainly of lowlands shaped by the glacial and denudative processes.

In this study, U-Net was used as a well-established reference model representing the convolutional neural network approach. Although it is widely used for image segmentation, it has some limitations related to the optimization of a huge number of parameters. Since its publication, an improved version has been proposed by Dinh et al.^57^, namely U-Lite, which requires fewer parameters (but still hundreds of thousands). There are also alternative CNN architectures with relatively fewer parameters, for example LeNet-5, requiring 60,000 parameters^58^ or its improved version (3DLeNet) recently proposed by Fırat et al.^59^ for classifying hyperspectral images. However, simplifying the architecture and reducing the number of parameters can make the model unable to recognize complex spatial patterns and structures, and therefore its effectiveness will still be low. The better performance of tree-based algorithms compared to CNNs in the study can be explained by the fact that they can perform better when handling few data observations. In a digital soil mapping experiment utilizing Random Forests, Bouslihim et al.^60^ showed that it could provide good performance by selecting only a few of the most relevant explanatory variables.

While this article focuses on the mapping of existing digital geomorphological maps using automatic classification on a regional scale, a further question arises: Do the methods and dataset used allow extrapolation of results for the entire country? In order to answer the question, we attempted to use the XGBoost classifier, which was trained on a large sample of over 3 million observations and has previously provided promising results (Table 2). Based on the experiment, we conclude that at this point the results are unsatisfactory and do not meet mapping standards. The main limitation in this case is the insufficient amount of reference materials, as they constitute approximately 3% of the country's coverage (over 9,000 km^2^), thus causing the trained model to be unable to recognize the same landforms in areas with different geomorphological characteristics. This issue was discussed more extensively by Bouasria et al.^61^ in the context of spatial extrapolation, where authors concluded that increasing the size of the spatial extent of the survey reduces the accuracy of the model. Therefore, we recommend further work to increase coverage by digital geomorphological maps at a scale of 1:100,000.

## Conclusions

In this article, we evaluated the potential of applying machine learning models and convolutional neural network to automatic geomorphological mapping and examined the usefulness and impact of selected geomorphometric variables on the results of landform classifications. Based on the results of this study, we can conclude that supervised learning methods are effective for mapping known sheets (Fig. 6), but ineffective for extrapolation to new areas, especially when the catalog of landforms is very extensive. Therefore, at this point, we can state that automatic methods cannot replace the traditional approach, but they can support mapping if the geomorphological characteristics in the predicted area are similar to those in the training dataset.

We used diagnostic techniques based on the analysis of the importance of geomorphometric variables to indicate the most useful variables for geomorphological mapping, and accumulated local effects plots to precisely examine how their values influence the model's classification decisions. This made the obscure and complicated classification mechanisms of the black box model more explicit and open to human interpretation.

The topic of automatic mapping remains unsolved, and further research is required. Further work should primarily focus on developing better geomorphometric variables for machine learning models and improving the architecture of the convolutional neural network to detect rarer landforms. In addition, future work should also address the issue of spatial validation at the model training and testing stages. Regarding the issue of explaining the decisions made by classification models, it would be useful to check the differences and similarities in the method inspired by game theory, i.e., *shapley additive explanation* proposed by Lundberg et al.^62^.

### Supplementary Information

Supplementary Figures.

## Data Availability

The programming scripts used for this analysis and to generate the figures are available in the following GitHub repository: https://github.com/kadyb/geomorph_classification. The reference geomorphological maps are available from the Head Office of Geodesy and Cartography in Poland, but restrictions apply to the availability of these data, which were used under license for the current study, and so are not publicly available.
