# Supplementary material for: Explanation of the influence of geomorphometric variables on the landform classification based on selected areas in Poland
Source: Sci Rep. 2024 Mar 5;14:5447. doi: 10.1038/s41598-024-56066-6 (PMC10914745; doi:10.1038/s41598-024-56066-6)
Supplement: Supplementary file 1 — Supplementary Figures. [file 41598_2024_56066_MOESM1_ESM.pdf]

Explanation of the influence of geomorphometric variables on the landform classification based on selected areas in Poland

Krzysztof Dyba

Supplementary Figure S1. Legend for the digital geomorphological map of Poland at a scale of 1:100,000. It has been adapted from the technical manual. The table includes only area forms. The first column contains the symbol, the second column contains the Polish translation, and the third column contains the English translation. For more details, see Rączkowska and Zwoliński (2015).

ENDOGENIC FORMS

N02	wzniesienie wysoczyzny morenowej związane z tektoniką solną	moraine plateau elevation due to salt tectonics

DENUDATIONAL FORMS

D01	powierzchnia zrównania w położeniu wierzchowinowym	planation surface in watershed setting
D02	powierzchnia zrównania w położeniu stokowym	planation surface in mid-slope setting
D03	powierzchnia zrównania w dnach kotlin	planation surface in basin setting
D09	czoło progu strukturalnego (kuesty)	cuesta face
D10	zaproże progu strukturalnego (kuesty)	cuesta backslope
D13	kotlina śródgórska	intramontane basin
D17	ostaniec denudacyjny - twardzielec	residual hill due to rock hardness
D18	ostaniec denudacyjny - nek	residual hill - volcanic neck
D19	ostaniec denudacyjny - góra stołowa	residual hill - mesa
D20	ostaniec denudacyjny - pozostałe	residual hill - others
D21	płaskowyż	plateau
D23	stok	slope surface
D24	ściana skalna lub stok skalny	rock wall or rock slope
D25	akumulacyjny stok gruzowy	depositional scree slope
D33	dolina erozyjno-denudacyjna	erosional-denudational valley

PERIGLACIAL FORMS

Y01	równina denudacji peryglacjalnej	bevelled surface of periglacial denudation
Y02	reliktowy lodowiec gruzowy	relict rock glacier

GLACIAL FORMS

G01	wysoczyzna morenowa płaska	flat moraine plateau
G02	wysoczyzna morenowa falista	undulating moraine plateau
G03	wysoczyzna ostańcowa	moraine remnant plateau
G04	wysoczyzna ostańcowa z pokrywą lessową	moraine remnant plateau covered by loess
G08	starsza forma przeobrażona subglacjalnie	overridden landform
G09	zagłębienie końcowe	terminal basin
G10	kocioł polodowcowy	cirque
G12	żłób polodowcowy	glacial trough
G14	misa polodowcowa	glacial overdeepening
G18	rynna polodowcowa	tunnel valley
G20	morena czołowa akumulacyjna	accumulative moraine
G21	morena czołowa spiętrzona	thrust moraine
G23	stożek glacimarginalny	ice-contact fan
G25	dno doliny z pokrywą fluwioglacjalną	glacial trough bottom with glacifluvial cover
G26	dno doliny z pokrywą morenową	glacial trough bottom with glacial debris cover
G27	morena pagórkowata	hummocky moraine
G28	morena martwego lodu	dead ice-moraine
G29	kem i stoliwo kemowe	kame and kame plateau
G30	terasa kemowa	kame terrace
G31	zagłębienie wytopiskowe	kettle hole
G32	stożek sandrowy/glacifluwialny	outwash fan
G33	równina lub szlak sandrowy	outwash plain or outwash track
G34	dolina proglacjalna	proglacial valley
G35	dolina marginalna	ice-marginal valley
G36	równina erozyjna wód roztopowych	meltwater erosional plain
G37	terasa pradolinna	ice-marginal valley terrace
G38	równina zastoiskowa	ice-dammed lake plain
G39	delta glacifluwialna	glacifluvial delta

FLUVIAL FORMS

F02	dolina wciosowa	v-shaped valley
F07	dno doliny płaskodennej rzeki anastomozującej	flat valley bottom of anastomosing river
F08	terasa zalewowa	floodplain
F09	terasy nadzalewowe	river terraces
F18	delta schowana	inverted delta
F19	stożek napływowy	alluvial fan
F20	równina proluwialna	proluvial plain

KARST FORMS

K01	depresja krasowa	karst depression

AEOLIAN FORMS

E01	ostaniec deflacyjny	wind eroded sandy hillock
E02	niecka deflacyjna	blowout
E03	wydmy ruchome	moving dunes
E05	zespół wydm ustabilizowanych	group of stabilised dunes
E06	równina zwydmiona	dune plain
E07	garb lessowy	loess hillock
E08	równina lessowa	loess plateau
E09	równina eolicznych piasków pokrywowych	plain of eolian coversands

LITTORAL AND LACUSTRINE FORMS

L05	plaża	beach
L06	wydmy nadbrzeżne	coastal dunes
L07	kosa lub mierzeja	spit or sandbar
L08	delta wsteczna	regressive delta
L09	równina nadmorska	coastal plain
L10	równina pojeziorna	lacustrine plain

ANTHROPEGNIC FORMS

A05	antropogeniczny zbiornik wodny	anthropogenic water reservoir

Supplementary Figure S2. Accumulated local effects plots for individual geomorphological map sheets. Note the different ranges of geomorphometric variable values on the X-axis as the sheets represent different morphogenetic areas (see Figure 1 and Figure 5 in the main publication). Note also the different probability ranges of landform classification (Y-axis) – some curves are very subtle. Entropy is expressed in thousands.

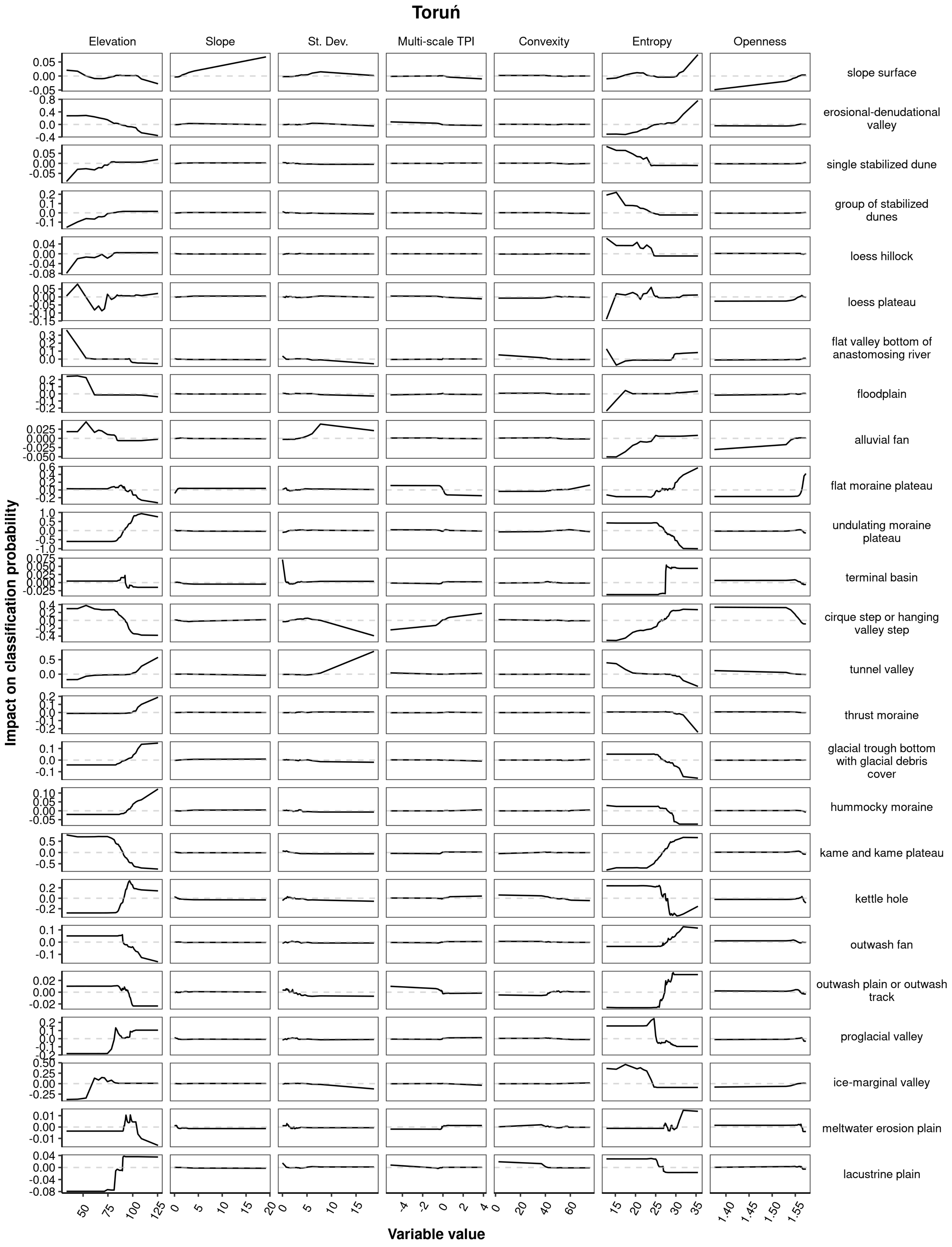

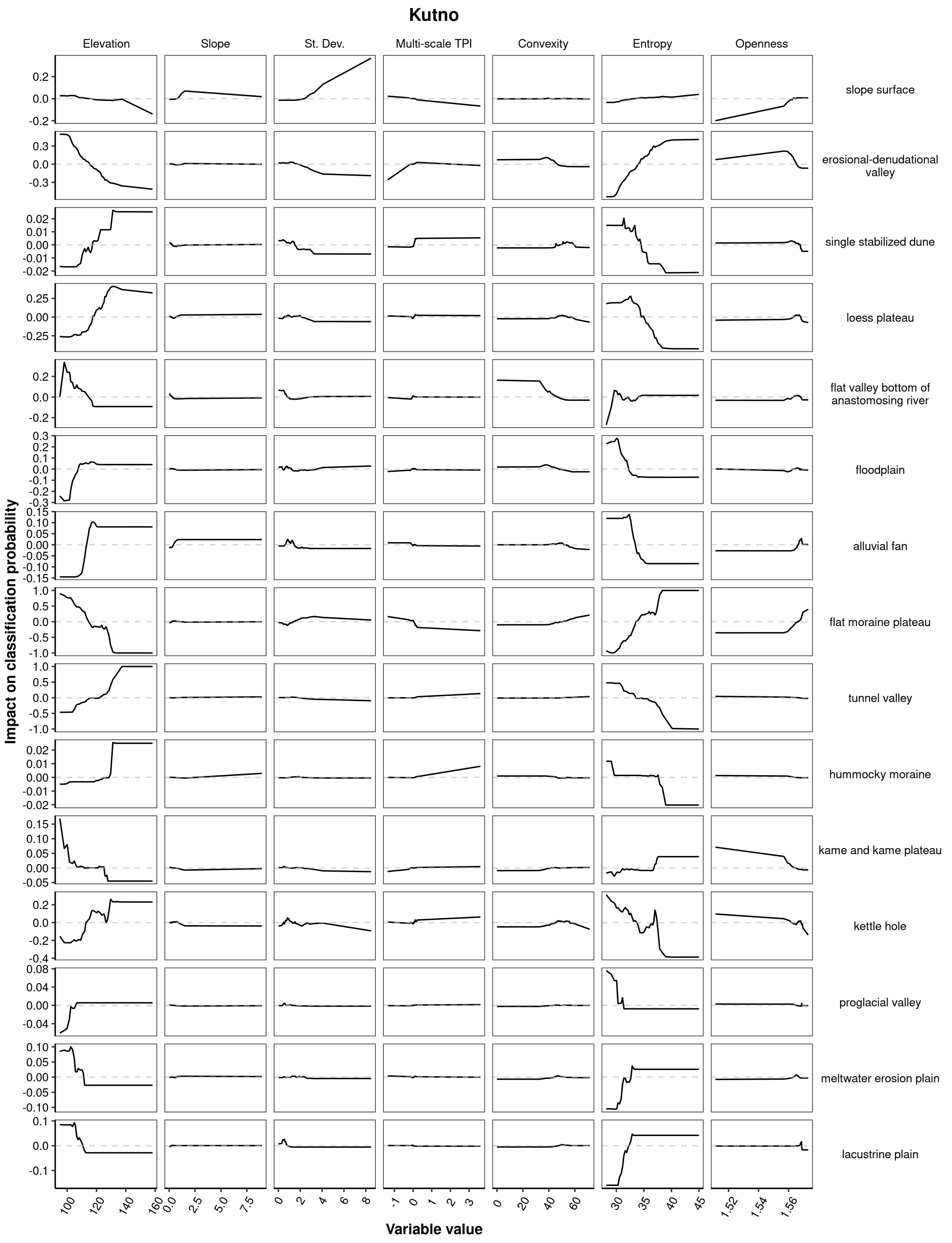
Kutno
Elevation
Slope
St. Dev.
Multi-scale TPI
Convexity
Entropy
Openness
slope surface
erosional-denudational valley
single stabilized dune
loess plateau
flat valley bottom of anastomosing river
floodplain
alluvial fan
flat moraine plateau
tunnel valley
hummocky moraine
kame and kame plateau
kettle hole
proglacial valley
meltwater erosion plain
lacustrine plain
Impact on classification probability
Variable value

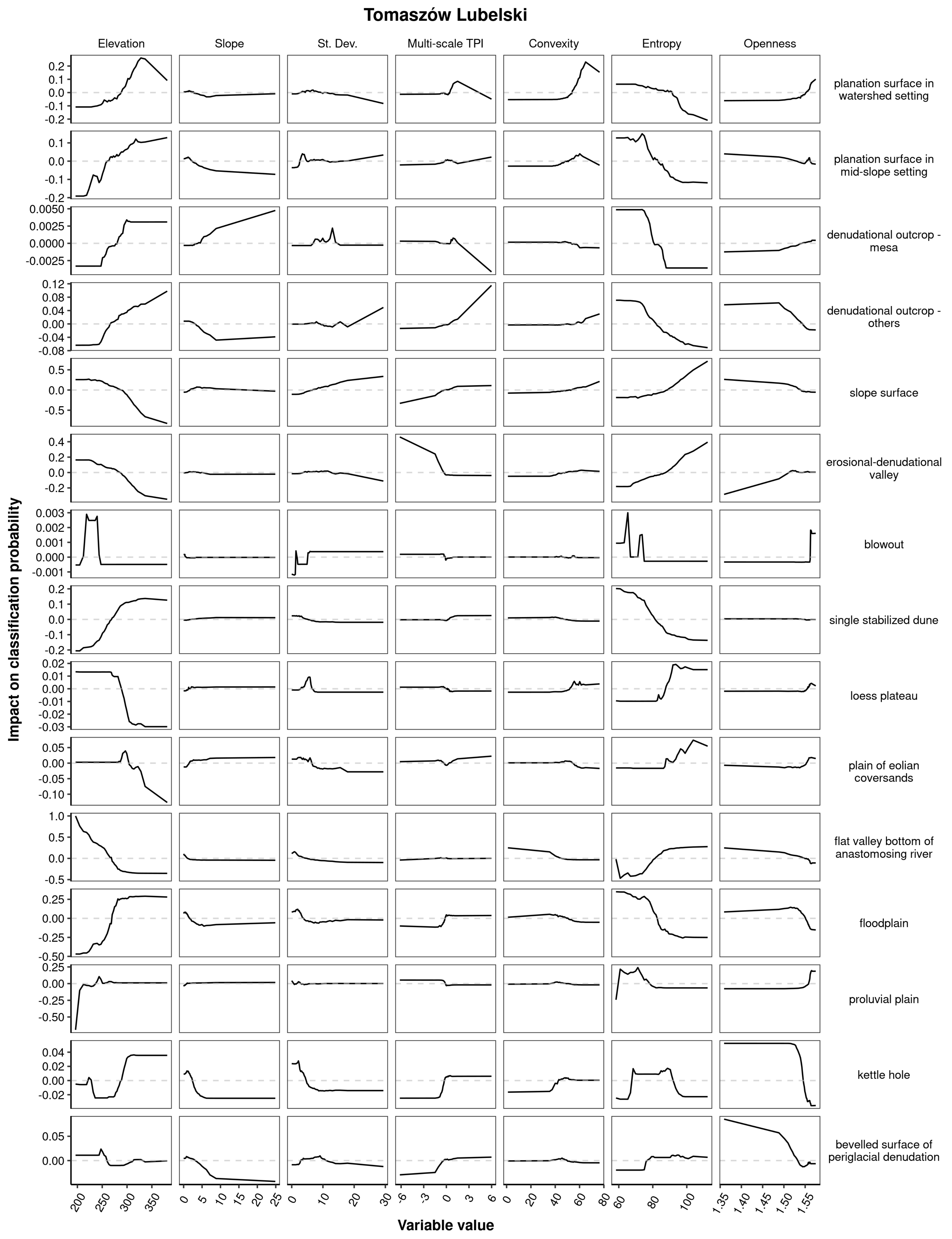
Tomaszów Lubelski
Elevation
Slope
St. Dev.
Multi-scale TPI
Convexity
Entropy
Openness
planation surface in watershed setting
planation surface in mid-slope setting
denudational outcrop - mesa
denudational outcrop - others
slope surface
erosional-denudational valley
blowout
single stabilized dune
loess plateau
plain of eolian coversands
flat valley bottom of anastomosing river
floodplain
proluvial plain
kettle hole
bevelled surface of periglacial denudation
Impact on classification probability
Variable value

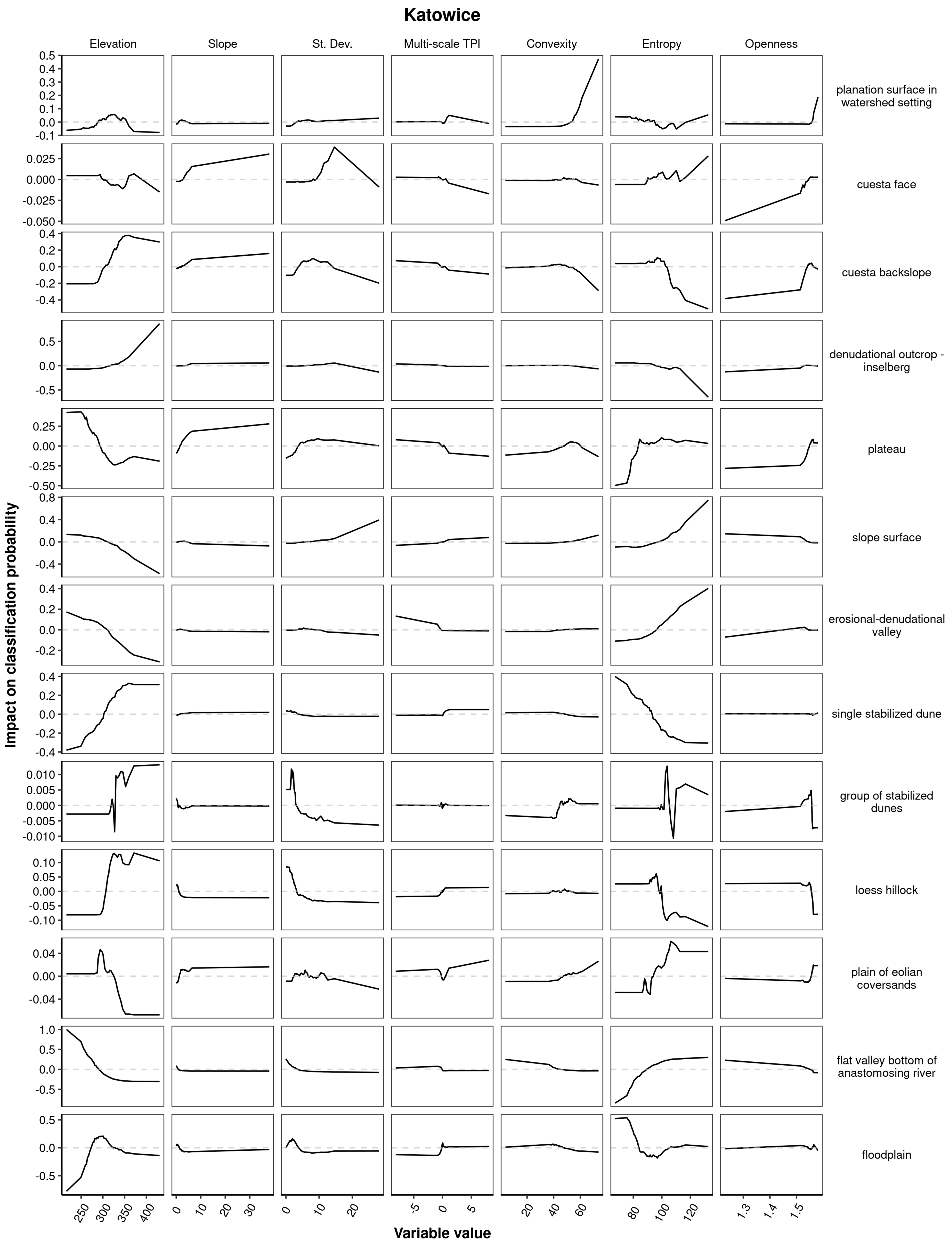

Katowice
Elevation
Slope
St. Dev.
Multi-scale TPI
Convexity
Entropy
Openness
planation surface in watershed setting
cuesta face
cuesta backslope
denudational outcrop - inselberg
plateau
slope surface
erosional-denudational valley
single stabilized dune
group of stabilized dunes
loess hillock
plain of eolian coversands
flat valley bottom of anastomosing river
floodplain
Impact on classification probability
Variable value

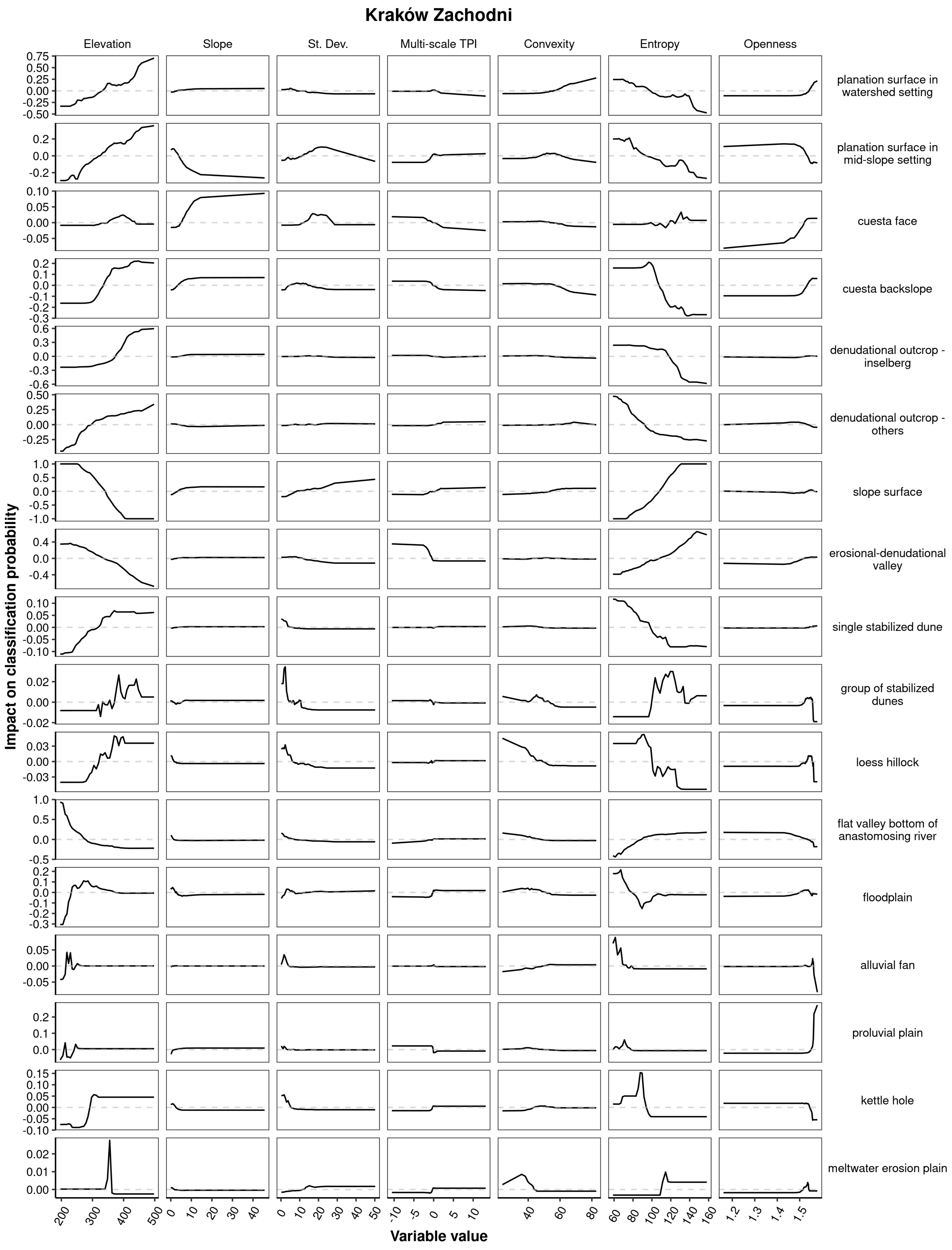
Kraków Zachodni
Elevation
Slope
St. Dev.
Multi-scale TPI
Convexity
Entropy
Openness
planation surface in watershed setting
planation surface in mid-slope setting
cuesta face
cuesta backslope
denudational outcrop - inselberg
denudational outcrop - others
slope surface
erosional-denudational valley
single stabilized dune
group of stabilized dunes
loess hillock
flat valley bottom of anastomosing river
floodplain
alluvial fan
proluvial plain
kettle hole
meltwater erosion plain
Impact on classification probability
Variable value

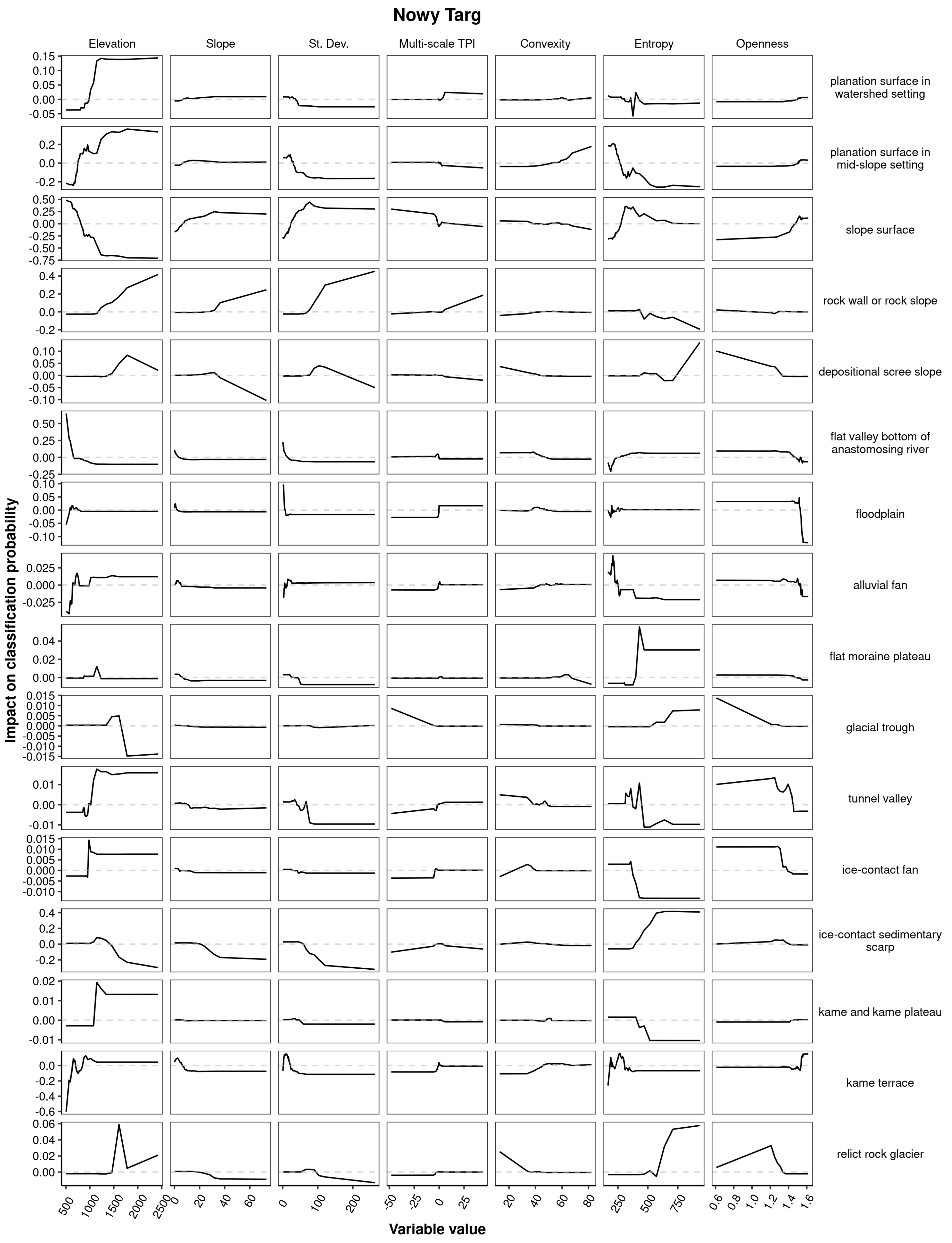
Nowy Targ
Elevation
Slope
St. Dev.
Multi-scale TPI
Convexity
Entropy
Openness
planation surface in watershed setting
planation surface in mid-slope setting
slope surface
rock wall or rock slope
depositional scree slope
flat valley bottom of anastomosing river
floodplain
alluvial fan
flat moraine plateau
glacial trough
tunnel valley
ice-contact fan
ice-contact sedimentary scarp
kame and kame plateau
kame terrace
relict rock glacier
Impact on classification probability
Variable value

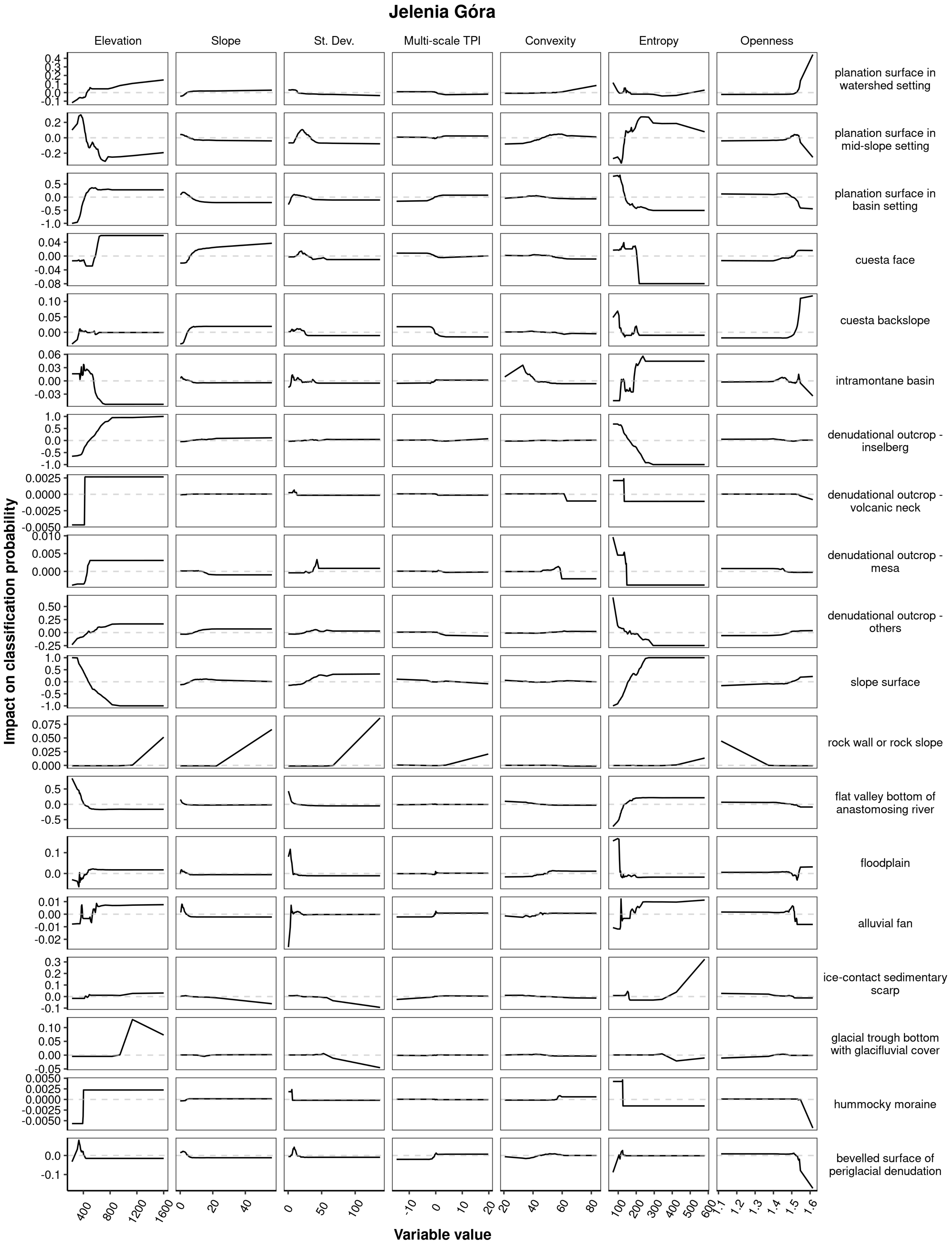
Jelenia Góra
Elevation
Slope
St. Dev.
Multi-scale TPI
Convexity
Entropy
Openness
planation surface in watershed setting
planation surface in mid-slope setting
planation surface in basin setting
cuesta face
cuesta backslope
intramontane basin
denudational outcrop - inselberg
denudational outcrop - volcanic neck
denudational outcrop - mesa
denudational outcrop - others
slope surface
rock wall or rock slope
flat valley bottom of anastomosing river
floodplain
alluvial fan
ice-contact sedimentary scarp
glacial trough bottom with glacifluvial cover
hummocky moraine
bevelled surface of periglacial denudation
Impact on classification probability
Variable value
